# The Involvement of Sirtuin 1 Dysfunction in High-Fat Diet-Induced Vascular Dysfunction in Mice

**DOI:** 10.3390/antiox11030541

**Published:** 2022-03-12

**Authors:** Ning Xia, Gisela Reifenberg, Christian Schirra, Huige Li

**Affiliations:** Department of Pharmacology, Johannes Gutenberg University Medical Center, Langenbeckstr. 1, 55131 Mainz, Germany; reifenb@uni-mainz.de (G.R.); christian.schirra@uk-augsburg.de (C.S.)

**Keywords:** perivascular adipose tissue (PVAT), sirtuin 1 (SIRT1), vascular function, nicotinamide phosphoribosyltransferase (NAMPT), nicotinamide adenine dinucleotide (NAD^+^) biosynthesis

## Abstract

High-fat diet (HFD)-induced vascular impairment in mice is associated with a dysfunction of the perivascular adipose tissue (PVAT). The present study was conducted to investigate the involvement of sirtuin 1 (SIRT1). Male C57BL/6J mice were fed an HFD for 20 weeks to induce obesity. Vascular function was analyzed using a wire myograph system. In obese mice, the vasodilator response of PVAT-containing aortas to acetylcholine was reduced, although the vascular function of PVAT-free aortas remained normal. SIRT1 activity in PVAT of obese mice was reduced despite enhanced SIRT1 expression. Nicotinamide adenine dinucleotide (NAD^+^) levels and the NAD^+^/NADH ratio in the PVAT of obese mice were decreased, which was likely attributable to a downregulation of the NAD^+^-producing enzyme NAMPT. The reduced SIRT1 activity was associated with an enhanced acetylation of the endothelial nitric oxide synthase (eNOS) in the PVAT. Ex vivo incubation of PVAT-containing aorta from obese mice with NAD^+^ led to a complete normalization of vascular function. Thus, reduced SIRT1 activity due to NAD^+^ deficiency is involved in obesity-induced PVAT dysfunction.

## 1. Introduction

Over the past few decades, the prevalence of obesity has become a major health challenge around the world [1]. Not only for many cancer types, but obesity is also a risk factor for cardiovascular disease and metabolic syndrome [2].

Obesity has numerous adverse effects on hemodynamics and cardiovascular structure and function [3]. In obese patients or patients with metabolic syndrome, vascular dysfunction is evident in both large conduits [4] and small arteries [5]. Patients with obesity have a higher risk of developing hypertension and stroke [3].

In addition to its structural supporting role for the blood vessels, perivascular adipose tissue (PVAT) is also involved in regulating vascular biology [6]. The nitric oxide (NO)-producing enzyme endothelial NO synthase (eNOS) is expressed in the PVAT, and PVAT eNOS plays an important role in regulating vascular function [7,8,9].

The eNOS function can be regulated by different signaling pathways [8]. The eNOS can be deacetylated by sirtuin 1 (SIRT1), a NAD^+^-dependent deacetylase. Deacetylation of the eNOS by SIRT1 increases the enzymatic activity of the eNOS [10]. In a previous study, we observed for the first time an enhanced eNOS acetylation in the PVAT of diet-induced obesity mice [11]. SIRT1 expression was not changed in PVAT by a high-fat diet (HFD) feeding. However, the expression of NAMPT, the rate-limiting enzyme for NAD^+^ biosynthesis, was reduced in the PVAT [11]. However, the NAD^+^ and SIRT1 activity could not be measured in that study, and causality between the reduced NAMPT expression and PVAT dysfunction could not be shown. 

The present study is an extension of our previous works [8,11] aiming to examine the significance of SIRT1 in the PVAT function. We show that the reduced expression of NAMPT leads to a decrease in NAD^+^ level and SIRT1 activity in PVAT in diet-induced obese mice. The reduced SIRT1 activity in the PVAT is due to a deficiency of NAD^+^ by HFD. Moreover, ex vivo incubation with exogenous NAD^+^ restores the PVAT function. 

## 2. Materials and Methods

### 2.1. Materials 

The NAD^+^ (β-Nicotinamide adenine dinucleotide hydrate) was purchased from Sigma-Aldrich (Taufkirchen, Germany). All other chemicals were of analytical grade and were obtained from Sigma-Aldrich (Taufkirchen, Germany), Fluka (Schwerte, Germany), or Merck (Darmstadt, Germany).

### 2.2. Animals

To induce obesity, male C57BL/6J mice from Janvier Labs (Le Genest-Saint-Isle, France) were used [12]. At the age of 6 weeks, the animals were randomly assigned into two groups to receive either the normal control diet (NCD, 11% energy from fat; ssniff, Soest, Germany) or HFD (60% energy from fat; ssniff^®^ EF D12492; ssniff, Soest, Germany) for 20 weeks.

The animals were housed in a temperature- and humidity-controlled room according to Good Laboratory Practice rules. The animal experiment was approved by the responsible regulatory authority (Landesuntersuchungsamt Rheinland-Pfalz; 23 177-07/G 17-1-020) and was conducted in accordance with the German animal protection law and the National Institutes of Health (NIH) Guide for the Care and Use of Laboratory Animals.

### 2.3. Measurement of Vascular Function

Thoracic aortas with or without the PVAT were taken for the measurement of vascular function with a wire myograph system (Danish Myo Technology, Aarhus, Denmark). Isometric tension was recorded and analyzed for the assessment of vascular function [7]. 

### 2.4. Gene Expression Analyses

RNA was isolated using peqGOLD TriFast™ (PEQLAB; Eralngen, Germany), and cDNA was generated with the High-Capacity cDNA Reverse Transcription Kit (Applied Biosystems; Waltham, MA, USA). Quantitative real-time RT-PCR (qPCR) reactions were performed on a StepOnePlus™ Real-Time PCR System (Applied Biosystems; Thermo Fisher Scientific, Waltham, MA, USA) using SYBR^®^ Green JumpStart™ Taq ReadyMix™ (Sigma-Aldrich, Taufkirchen, Germany) and 20 ng cDNA. Relative mRNA levels of target genes were quantified using comparative threshold C_T_ normalized to housekeeping gene TATA-binding protein (TBP; accession number NM_013684) [13,14]. The mRNA expression in control animals with NCD was set at 100%. The following target genes were studied: SIRT1 (accession number NM_019812), nicotinamide phosphoribosyltransferase (NAMPT; NM_021524), Parp1 (NM_007415), and Parp2 (NM_009632). The qPCR primer sequences were as follows: SIRT1_forward: GCC AAA CTT TGT TGT AAC CCT GTA; SIRT1_reverse: TGG TGG CAA CTC TGA TAA ATG AA; NAMPT_forward: TTC CCG AGG GCT CTG TCA; NAMPT_reverse: GTA GCA CTC TGG GTC TGT GTT TTC; PARP1_forward: CAT GGT GTC CAA AAG TGC AAA CT; PARP1_reverse: GCA ACC TCT CCC AGC AGT ATT AA; PARP2_forward: CCA GCC CTG CCC ACT TC; PARP2_reverse: CCT GTG TCA CTT GCT GGT CCT A; TBP_forward: CTT CGT GCA AGA AAT GCT GAA T; TBP_reverse: CAG TTG TCC GTG GCT CTC TTA TT.

### 2.5. Western Blot Analyses

Total protein samples (30 µg each) from the aorta or the PVAT were used for Western blot analyses. The following primary antibodies were used: mouse monoclonal antibody against the eNOS (BD Transduction Laboratories; Cat# 610297); rabbit polyclonal antibody against SIRT1 (LifeSpan BioSciences; Cat# LS-B1564), rabbit polyclonal antibody against NAMPT (Aviva Systems Biology, Cat# ARP42255-T100), and mouse monoclonal antibody against β-tubulin (Sigma-Aldrich, Cat# T7816). 

### 2.6. Measurement of SIRT1 Activity in PVAT

The PVAT SIRT1 activity in homogenates was measured using CycLex SIRT1/Sir2 Deacetylase Fluorometric Assay Kit from MBL international Corporation (Woburn, MA, USA) according to the manufacturer’s instructions.

### 2.7. Measurement of NAD^+^ and NADH Levels in PVAT

The levels of NAD^+^ and NADH were measured in PVAT homogenates using CycLex^®^ NAD^+^/NADH Colorimetric Assay Kit from MBL international Corporation (Woburn, MA, USA) according to the manufacturer’s instructions.

### 2.8. Analysis of eNOS Acetylation

The PVAT tissue samples were ground to a fine powder in liquid nitrogen with a mortar, then lysis buffer (Thermo Fisher Scientific; Cat# 610297) was added to obtain the PVAT lysate. For performing the eNOS immunoprecipitation (IP), 1 mg PVAT lysate was incubated with 3 µg of the specific eNOS antibody (LifeSpan BioSciences; Cat# LS-C288673) for IP at 4 °C for 1 h. After that, the lysate was incubated with 50 μL Protein A/G Magnetic Beads at room-temperature for 1 h. The immunoprecipitates were boiled in a loading buffer for 5 min at 95 °C before being subjected to SDS/PAGE. Anti-acetylated lysine (Cell Signaling Technology; Cat# 9441) and the anti-eNOS (BD Transduction Laboratories; Cat# 610297) antibodies were used for immunoblotting, respectively. 

### 2.9. Statistical Analysis

Student’s *t*-test was used for a comparison of two groups. A two-way ANOVA was used to compare the curves. Results are presented as mean ± SEM. The *p* values < 0.05 were considered significant. A GraphPad Prism (GraphPad Prism 8.4.2, La Jolla, CA, USA) was used for statistical analysis.

## 3. Results

### 3.1. Involvement of PVAT in Obesity-Induced Vascular Dysfunction

**Figure 1:**
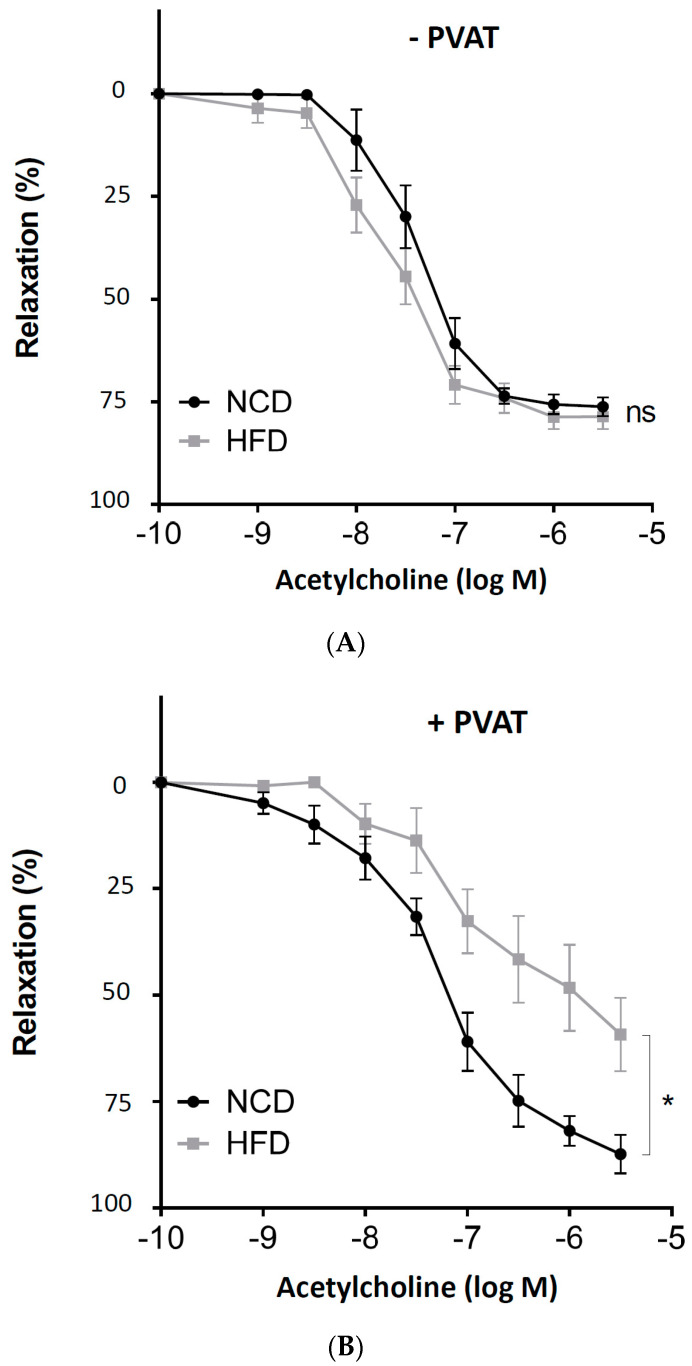
The role of perivascular adipose tissue (PVAT) in obesity-induced vascular dysfunction. Male C57BL/6J mice were treated with normal control diet (NCD) or high-fat diet (HFD) for 20 weeks. Aortic rings without (**A**) or with PVAT (**B**) were used for vascular function assessment with a wire myograph system. The rings were precontracted with noradrenaline, followed by relaxation in response to increasing concentrations of acetylcholine. Symbols represent mean ± SEM. ns, not significant; * *p* < 0.05; *n* = 8.

To induce obesity, male C57BL/6J mice were randomly allocated to two groups. At the age of 6 weeks, the HFD feeding was started. After 20 weeks of feeding, adiposity was evident (Table 1). Aortas with or without PVAT were prepared from mice for vascular function assessment with a wire myograph system. In the PVAT-free aortas from obese mice, the vasodilator response to acetylcholine remained normal (Figure 1A). In contrast, acetylcholine-induced vasodilation was clearly reduced in the aortas of obese mice, when the PVAT was left intact (Figure 1B).

### 3.2. Enhanced SIRT1 Expression, but Reduced SIRT1 Activity in PVAT of Obese Mice

**Figure 2:**
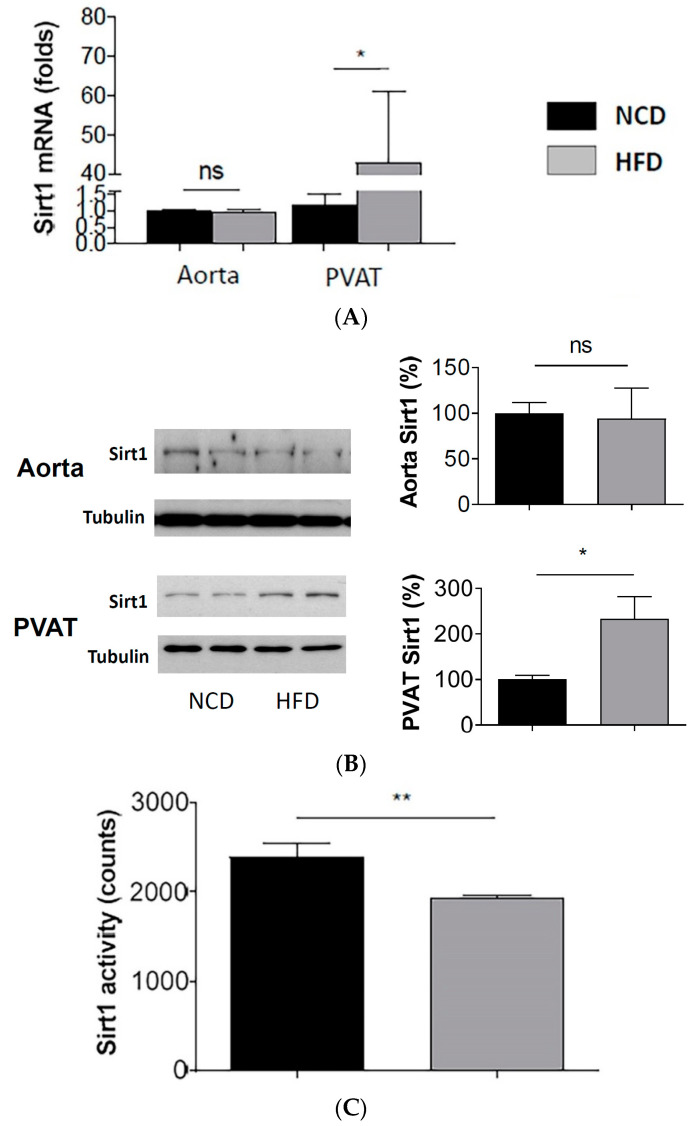
Reduced SIRT1 activity despite enhanced SIRT1 expression in PVAT of diet-induced obese mice. Male C57BL/6J mice were treated with normal control diet (NCD) or high-fat diet (HFD) for 20 weeks. The mRNA expression of SIRT1 (**A**) was studied with qPCR. The protein expression of SIRT1 (**B**) was studied with Western blot. The blots shown are representative for 3 independent experiments with similar results. * *p* < 0.05; ns, not significant; *n* = 10. SIRT1 activity (**C**) in mouse PVAT lysate was measured with SIRT1 deacetylase fluorometric assay kit. Columns represent mean ± SEM. ** *p* < 0.01; *n* = 10.

The mRNA (Figure 2A) and protein (Figure 2B) expression of SIRT1 were not changed in the aorta, but significantly enhanced in the PVAT of diet-induced obese mice. Unexpectedly, the enhanced SIRT1 expression in the PVAT was associated with reduced SIRT1 activity (Figure 2C).

### 3.3. Reduced NAMPT Expression, NAD^+^ Level and NAD^+^/NADH Ratio in PVAT of Diet-Induced Obese Mice 

**Figure 3:**
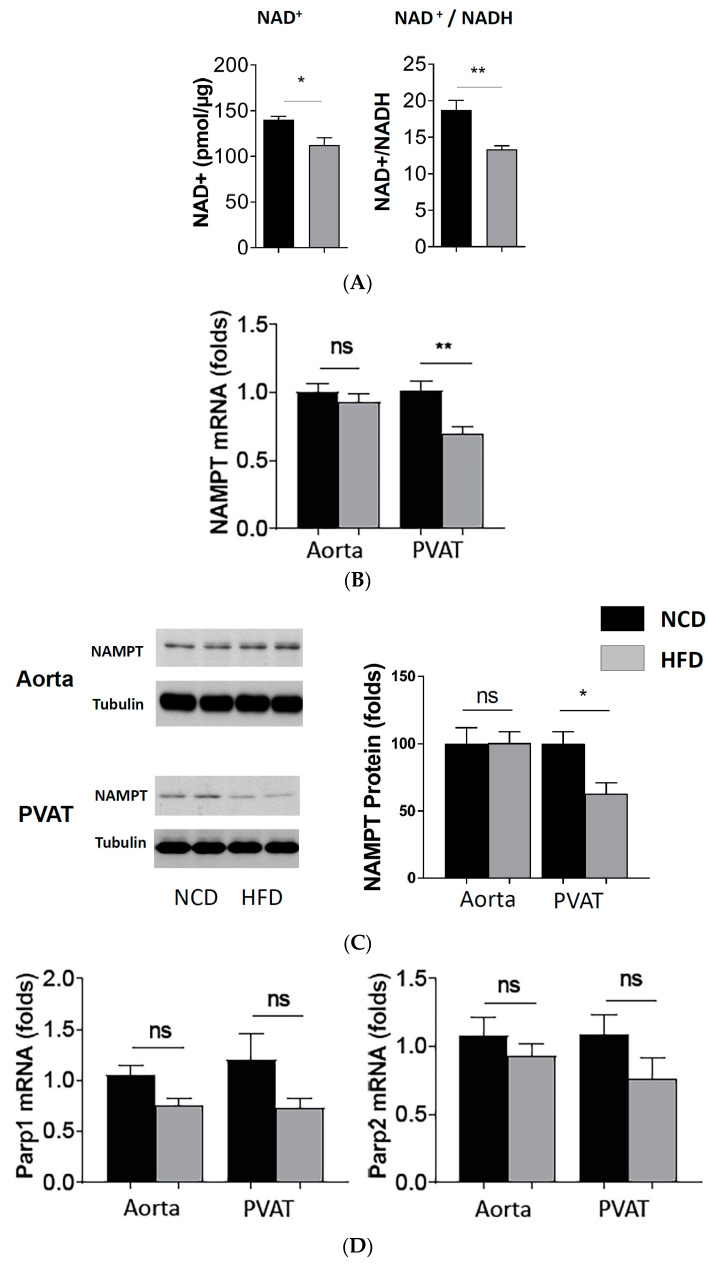
Reduced NAD^+^ level, NAD^+^/NADH ratio and NMAPT expression in PVAT of diet-induced obese mice. Male C57BL/6J mice were treated with NCD or HFD for 20 weeks. NAD^+^ level and NADH in mouse PVAT lysate were measured with NAD^+^/NADH Colorimetric Assay kit (**A**). Columns represent mean ± SEM. * *p* < 0.05, ** *p* < 0.01, *n* = 6. The mRNA expression of NAMPT (**B**) was studied with qPCR. Columns represent mean ± SEM. ns, not significant; ** *p* < 0.01, *n* = 6. The protein expression of NAMPT (**C**) was studied with Western blot. The blots shown are representative for 3 independent experiments with similar results. ns, not significant; * *p* < 0.05; *n* = 10. Parp1 and Parp2 expression (**D**) was studied with (qPCR). *n* = 10. ns, not significant.

The NAD^+^ level and the NAD^+^/NADH ratio were found to have decreased in the PVAT of HFD mice (Figure 3A). The mRNA (Figure 3B) and protein (Figure 3C) expression of the NAMPT were not changed in the aorta of HFD-fed mice. However, they are both reduced in the PVAT of HFD-fed mice. The NAMPT is a rate-limiting enzyme for NAD^+^ biosynthesis. In contrast, the expression level of PARP1 and PARP2, two NAD^+^-consuming enzymes, was not changed in the PVAT of diet-induced obese mice (Figure 3D).

### 3.4. Enhanced eNOS Acetylation Level in the PVAT of Diet-Induced Obese Mice

**Figure 4:**
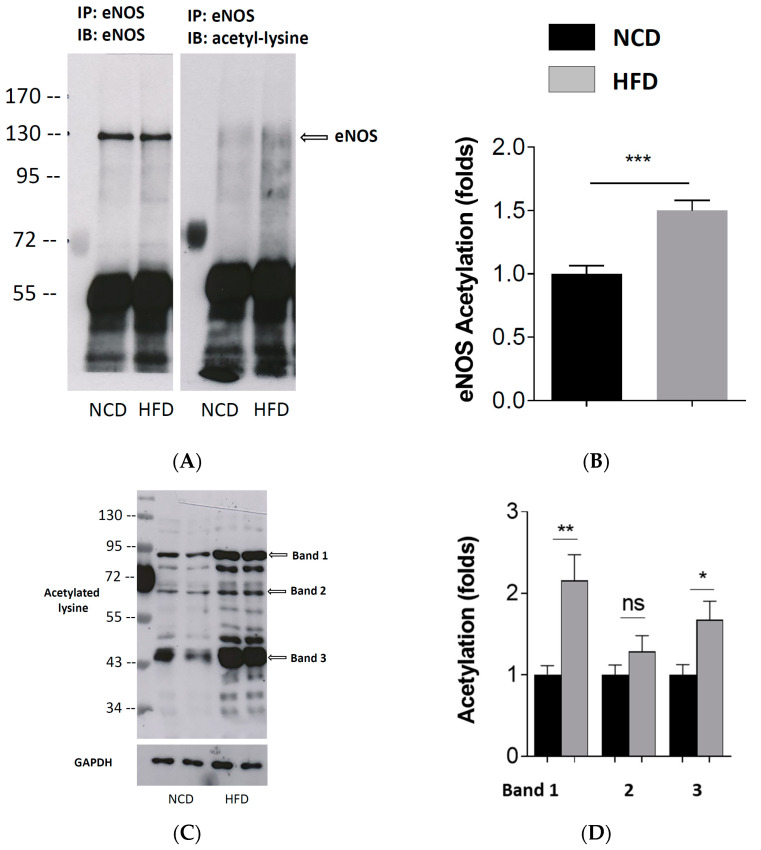
Enhanced eNOS acetylation in PVAT of diet-induced obese mice. Male C57BL/6J mice were treated with NCD or HFD for 20 weeks. eNOS immunoprecipitation (IP) was performed with the PVAT samples using an eNOS antibody. Immunoblotting (IB) was performed with the eNOS IP product using an antibody against eNOS and an antibody against acetyl-lysine, respectively. The band detected with the antibody against acetyl-lysine at the level of eNOS was considered acetyl-eNOS. The blots shown (**A**) are representative of 3 independent experiments with similar results. Results of densitometric analyses for the acetyl-eNOS normalized to a total eNOS are shown in (**B**). Columns represent mean ± SEM. *** *p* < 0.001, *n* = 6. In separate experiments, Western blot analyses (WB) were performed with an antibody against acetyl-lysine using the PVAT samples without IP. The blots shown (**C**) are representative of 3 independent experiments with similar results. The results of densitometric analyses for acetylated proteins normalized to GAPDH are shown in (**D**). Columns represent mean ± SEM. ns, not significant; * *p* < 0.05, ** *p* < 0.01, *n* = 10.

The eNOS immunoprecipitation (IP) was performed with the PVAT samples using a specific eNOS antibody. Immunoblotting (IB) was performed with the IP product using an antibody against the eNOS and an antibody against acetyl-lysine, respectively. The band detected with the antibody against acetyl-lysine at the level of eNOS was considered acetyl-eNOS. As shown in Figure 4, the HFD-feeding enhanced the acetylation of the eNOS (Figure 4A,B). If IB was performed without the eNOS IP, a number of acetyl protein bands were detected. The HFD led to enhanced acetylation of diverse proteins (Figure 4C,D) in the PVAT. 

### 3.5. NAD^+^ Incubation Restores PVAT Function

**Figure 5:**
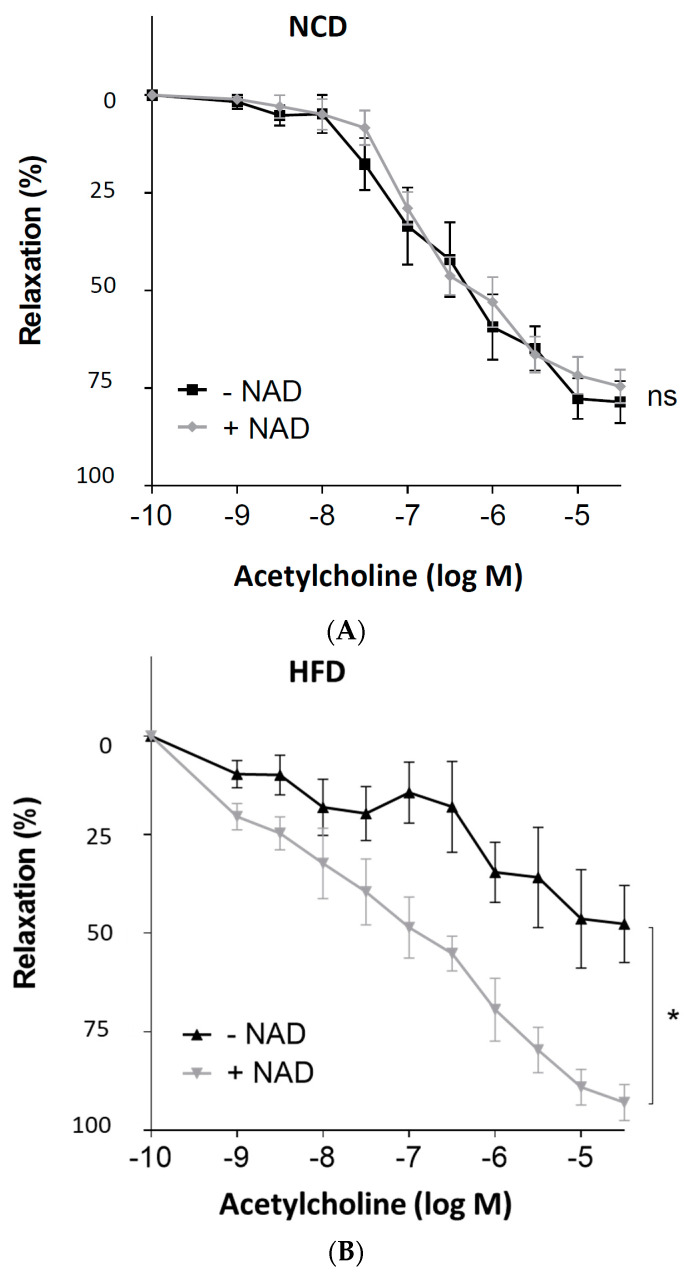
Improvement of the PVAT function by exogenous NAD^+^ in diet-induced obese mice. Male C57BL/6J mice were treated with (**A**) NCD or (**B**) HFD for 20 weeks. The PVAT-containing aortas were isolated and incubated in the myograph system with 350 μmol/L NAD^+^ for 6 h. Then, the vessels were contracted with noradrenaline before acetylcholine was added to induce vasodilation. Symbols represent mean ± SEM. * *p* < 0.05, *n* = 6. ns, not significant.

Male C57BL/6J mice were treated with NCD or HFD for 20 weeks. The PVAT-containing aortas were isolated and incubated in the myograph system with 350 μmol/L NAD^+^ for 6 h. Then, the vessels were contracted with noradrenaline before the acetylcholine was added to induce vasodilation. As shown in Figure 5, ex vivo incubation of the PVAT-containing aortas from HFD mice with NAD^+^ led to a complete normalization of vasodilator response to acetylcholine.

## 4. Discussion

In the present study, we show that HFD-induced vascular dysfunction is only evident in PVAT-containing but not in PVAT-free aorta. This is consistent with our previous study [7] demonstrating the important role of PVAT in obesity-induced vascular dysfunction. In another study, we reported that PVAT dysfunction in obesity was associated with an enhanced eNOS acetylation and reduced the eNOS activity [11]. We proposed that the enhanced eNOS acetylation was due to reduced SIRT1 activity; but the SIRT1 activity could not be analyzed in that study [11]. The present study is a continuation and extension of our previous studies. Major novel findings of the present study include: (i) HFD-feeding in mice leads to a downregulation of the NAD^+^-producing enzyme NAMPT resulting in a reduction in the NAD^+^ level and the NAD^+^/NADH ratio in the PVAT; (ii) The SIRT1 activity in the PVAT of obese mice is reduced despite an enhanced SIRT1 expression; (iii) The PVAT (and vascular) dysfunction of DIO mice can be normalized by an ex vivo incubation of the PVAT-containing aorta with NAD^+^, indicating a causal role of NAD^+^ deficiency in HFD-induced PVAT dysfunction. 

PVAT has attracted much interest since the discovery of its anti-contractile effect in 1991 [6]. It is now well-established that PVAT has pivotal roles in regulating vascular function by producing a large number of biologically active factors. These molecules, including NO, hydrogen sulfide, hydrogen peroxide, chemokines, cytokines, and adipokines, can exert effects on the vascular system through both paracrine and endocrine mechanisms [8,15,16]. Nevertheless, molecular mechanisms concerning how PVAT contributes to vascular physiology and pathophysiology are still not completely understood and PVAT remains a fascinating research topic.

The enzyme eNOS is named after the cell type in which it was discovered. However, eNOS is not only expressed in endothelial cells. We have shown that eNOS is present in the PVAT and eNOS in PVAT is functionally active [7]. Diet-induced obesity leads to PVAT eNOS dysfunction. Pharmacological improvement of the PVAT eNOS function, either by ex vivo incubation with L-arginine and arginase inhibitors [7], or by in vivo treatment of the obese mice with the standardized Crataegus extract WS1442 [11], restores PVAT function, indicating a crucial role of eNOS in maintaining PVAT function. 

**Figure 6:**
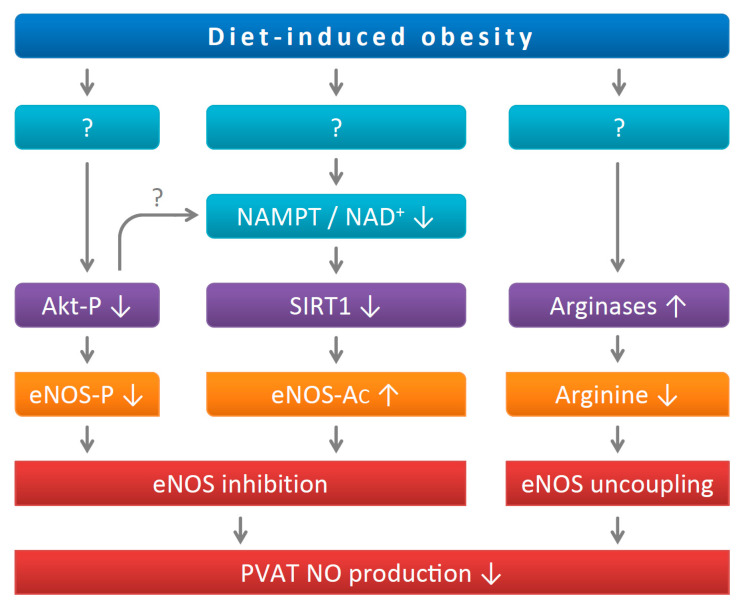
PVAT eNOS dysregulation in diet-induced obese mice. Diet-induced obesity leads to inhibition of Akt phosphorylation. The downregulation of NAMPT reduces cellular NAD^+^ levels and SIRT1 activity. The resulting reduction in eNOS phosphorylation and enhancement of eNOS acetylation both decrease eNOS activity. In addition, HFD feeding causes eNOS uncoupling due to arginine deficiency because of arginase induction. The illustrated mechanisms are a summary of results from the present study and two previous publications [7,11].

The activity and function of the eNOS are regulated by different mechanisms, including post-translational modifications, such as phosphorylation and acetylation [17] (Figure 6). The eNOS is a direct target of SIRT1 and deacetylation of eNOS by SIRT1 enhances eNOS activity [10]. In a previous study, we have observed an enhanced eNOS acetylation in the PVAT of diet-induced obese mice [11], which is confirmed in the present study. Moreover, this is likely to be attributable to a dysregulation of SIRT1 activity in the PVAT. 

SIRT1 is expressed in the vascular wall and the PVAT plays a critical role in vasoprotection by regulating a myriad of signaling pathways [18]. In our previous study [11], the SIRT1 expression was not changed by HFD-feeding in the PVAT or in the aorta. In the present study, we found the SIRT1 expression was not changed in the aorta but enhanced in the PVAT (Figure 2). This discrepancy may be due to the difference in the experimental setting. In the 2017 study [11], the mice were treated with 45% HFD starting at the age of 8 weeks, while, in the present study, the animals were fed 60% HFD from the age of 6 weeks. Nevertheless, both studies show consistently that the expression of the NAD^+^-producing enzyme NAMPT was not changed in the aorta but reduced selectively in the PVAT (Figure 3).

NAD^+^ can be biosynthesized from L-tryptophan. In addition to this de novo biosynthesis, NAD^+^ can also be produced from the salvage pathway. The latter is considered the main source of NAD^+^ [19]. NAMPT plays an important role in NAD^+^ biosynthesis because it is the rate-limiting enzyme in the salvage pathway of NAD^+^ production. Close correlations have been found between the NAMPT and NAD^+^ levels [19,20]. Importantly, an enhanced expression of NAMPT not only increases NAD^+^ biosynthesis, but also reduces the levels of the SIRT1 inhibitor NAM (a precursor for NAD^+^ biosynthesis). Both mechanisms contribute to the increased SIRT1 activity by NAMPT [19,20].

NAD^+^ is not only consumed by SIRT1 as a co-factor, it is also utilized by PARP enzymes [21]. PARP1 and PARP2 are the most important PARP members and account for nearly all the cellular PARP activity [19]. They are ubiquitously expressed nuclear proteins involved in DNA repair and genomic stability. NAD^+^ is a required PARP activity and an overactivation of PARP may deplete the stores of cellular NAD^+^ [19]. By competing with the SIRT1 for NAD^+^, PARP1 is known to inhibit SIRT1 activity. PARP2, on the other hand, can also decrease the SIRT1 transcription [21]. In the present study, the expression of PARP1 and PARP2 was not changed by the HFD-feeding, indicating that the reduced SIRT1 activity was more likely a result of the decreased NAD^+^ level because of NAMPT downregulation.

Reduced NAD^+^ levels have been observed in the liver and white adipose tissue of HFD-treated mice [22]. In contrast, exercise [21,23] and calorie restriction (CR) [24] can increase tissue NAD^+^ levels. The molecular mechanisms underlying the downregulation of NAMPT in HFD-treated mice are still unclear. It was supposed that inflammation and/or oxidative stress may be responsible for the reduction in NAMPT-mediated NAD^+^ biosynthesis in the liver [21]. A recent study has shown that Akt activation by insulin increases the NAMPT expression in human breast cancer MCF-7 cells [25]. In our previous study, the downregulation of NAMPT expression in the PVAT of HFD mice was associated with a reduction in Akt phosphorylation [11]. Moreover, treatment of HFD mice with the standardized *Crataegus* special extracts WS1442 restores Akt phosphorylation and NAMPT expression in the PVAT [11]. Thus, it is conceivable that Akt may be a regulator of NAMPT expression in the PVAT. Nevertheless, more evidence is needed to be conclusive.

Not only is the absolute NAD^+^ level crucial for SIRT1 activity, but because NADH can act as a competitive inhibitor, the NAD^+^/NADH ratio is also considered highly relevant for SIRT1 activity [26]. In the present study, both the NAD^+^ level and the NAD^+^/NADH ratio were reduced, and both were likely to contribute to the reduced SIRT1 activity in the PVAT.

Raised NAD^+^ levels by niacin and other NAD^+^ precursors, such as NMN and NR, can activate SIRT1 in mice [21]. In the present study, we demonstrate that incubation of the PVAT-containing aortas with exogenous NAD^+^ restores the vascular function of DIO mice. This indicates that the reduced NAD^+^ level is a mechanism causally involved in obesity-induced vascular dysfunction. Moreover, our results demonstrate that vascular dysfunction in obesity is reversible. Thus, enhancing SIRT1 activity by improving the NAD^+^ level may represent a therapeutic strategy for the treatment of obesity-associated vascular complications.

In summary, the present study demonstrates that diet-induced obesity leads to vascular dysfunction, which is associated with reduced SIRT1 activity in PVAT despite (compensatorily) enhanced SIRT1 expression. The NAD^+^ and NAD^+^/NADH ratios are reduced in the PVAT of DIO mice, very likely due to a downregulation of the NAD^+^-producing enzyme NAMPT. Vascular dysfunction of DIO mice can be normalized by ex vivo incubation with NAD^+^, indicating a causal role of NAD^+^ deficiency in obesity-induced vascular dysfunction and the therapeutic potential of stimulating SIRT1 activity by improving NAD^+^ levels. Results from this and previous studies collectively demonstrate that eNOS is a key regulator of PVAT function (Figure 6).

## 5. Conclusions

SIRT1 dysfunction due to NAD^+^ deficiency is causally involved in obesity-induced PVAT dysfunction. Improving SIRT1 activity by elevating NAD^+^ levels may represent a novel therapeutic strategy for obesity-associated vascular disease.

## Figures and Tables

**Table 1:** Effect of dietary treatment on body weight and adiposity.

	NCD	HFD	*p* Value
BW (g)	31.35 ± 0.56	46.25 ± 0.64	*p* < 0.001
EAT (mg)	720.4 ± 43.8	2119.0 ± 64.0	*p* < 0.001
EAT/BW-ratio (mg/g)	22.86 ± 1.16	45.95 ± 1.44	*p* < 0.001

Data are expressed as mean ± SEM of 20 animals per group. NCD indicates normal control diet; HFD, high-fat diet; BW, body weight; EAT, epididymal adipose tissue.

## Data Availability

The data are contained within the article.

## Abbreviations

eNOS	endothelial nitric oxide synthase
HFD	high-fat diet
NAD	nicotinamide adenine dinucleotide
NAMPT	nicotinamide phosphoribosyltransferase
NCD	normal control diet
NO	nitric oxide
qPCR	Quantitative real time RT-PCR
PARP1	Poly [ADP-ribose] polymerase 1
PARP2	Poly [ADP-ribose] polymerase 2
PVAT	perivascular adipose tissue
SIRT1	sirtuin 1
TBP	TATA-binding protein
